# Rapid Diagnostic Algorithms as a Screening Tool for Tuberculosis: An Assessor Blinded Cross-Sectional Study

**DOI:** 10.1371/journal.pone.0049658

**Published:** 2012-11-21

**Authors:** Franz Ratzinger, Harald Bruckschwaiger, Martin Wischenbart, Bernhard Parschalk, Delmiro Fernandez-Reyes, Heimo Lagler, Alexandra Indra, Wolfgang Graninger, Stefan Winkler, Sanjeev Krishna, Michael Ramharter

**Affiliations:** 1 Division of Infectious Diseases and Tropical Medicine, Department of Medicine I, Medical University Vienna, Vienna, Austria; 2 Department of Laboratory Medicine, Medical University Vienna, Vienna, Austria; 3 Department of Internal Medicine, Krankenhaus der Barmherzigen Brüder, Vienna, Austria; 4 Information Systems Group, Institute of Bioinformatics, Johannes Kepler University, Linz, Austria,; 5 Division of Parasitology, National Institute for Medical Research, London, United Kingdom; 6 Austrian Agency for Health and Food Safety, Vienna, Austria; 7 Centre for Infection, St. George’s University of London, London, United Kingdom; 8 Institute for Tropical Medicine, University of Tübingen, Tübingen, Germany; Institut de Pharmacologie et de Biologie Structurale, France

## Abstract

**Background:**

A major obstacle to effectively treat and control tuberculosis is the absence of an accurate, rapid, and low-cost diagnostic tool. A new approach for the screening of patients for tuberculosis is the use of rapid diagnostic classification algorithms.

**Methods:**

We tested a previously published diagnostic algorithm based on four biomarkers as a screening tool for tuberculosis in a Central European patient population using an assessor-blinded cross-sectional study design. In addition, we developed an improved diagnostic classification algorithm based on a study population at a tertiary hospital in Vienna, Austria, by supervised computational statistics.

**Results:**

The diagnostic accuracy of the previously published diagnostic algorithm for our patient population consisting of 206 patients was 54% (CI: 47%–61%). An improved model was constructed using inflammation parameters and clinical information. A diagnostic accuracy of 86% (CI: 80%–90%) was demonstrated by 10-fold cross validation. An alternative model relying solely on clinical parameters exhibited a diagnostic accuracy of 85% (CI: 79%–89%).

**Conclusion:**

Here we show that a rapid diagnostic algorithm based on clinical parameters is only slightly improved by inclusion of inflammation markers in our cohort. Our results also emphasize the need for validation of new diagnostic algorithms in different settings and patient populations.

## Introduction

Tuberculosis is causing an estimated 1.7 million deaths per year and the highest burden of disease is found in regions of high HIV prevalence. [1] One of the main obstacles to effective treatment and control of tuberculosis is a lack of accurate, rapid, point-of-care and low-cost diagnostic tools. [2] Radiology and microscopy of sputum samples remain the most important diagnostic tools in low-income regions and culture, PCR, histology, and radiology are additional valuable diagnostic tools in high-income countries. Recently, the development of automated molecular tests for the diagnosis of pulmonary tuberculosis showed promising results, however this diagnostic approach is less useful for extra-pulmonary infections. [3] To date no diagnostic method is therefore able to provide high diagnostic accuracy in a timely manner for pulmonary and extra-pulmonary tuberculosis. Other diagnostic tools including the Mendel Mantoux skin test or interferon gamma release assays cannot reliably discriminate between latent infection and active disease. [4], [5] PCR based diagnostic tools are rapid and show promising diagnostic accuracy in sputum positive tuberculosis, however cost and extrapulmonary infections are limiting its usefulness.[6]–[8] Recently developed FACS based diagnostic methods for extra-pulmonary tuberculosis show promising diagnostic results but necessitate advanced technical equipment and skills, and wait for prospective evaluation in different patient populations. [9] Similarly, current efforts to identify novel biomarkers or screening rules for tuberculosis have not yet resulted in a reliable candidate molecule for further clinical assessment.[10]–[14].

Based on proteomic fingerprinting of serum Agranoff and colleagues proposed a rapid screening test for active tuberculosis based on the measurement of inflammation parameters including C-reactive protein, transthyretin, serum amyloid A, and neopterin. [15] The proposed classification-model was established by machine learning methods to obtain the best diagnostic algorithm. [16], [17] In that publication a diagnostic accuracy of up to 84% was reported in a prospectively obtained data set for the detection of active cases of primarily pulmonary tuberculosis. Although this test performance is far from perfect, a reliable algorithm might considerably help in classifying patients in high or low probability for tuberculosis. This might help to focus more time consuming and resource intensive investigations only on persons with high pre-test probability. To better appreciate the diagnostic potential of the previously published algorithm external validity needs to be assessed in different patient populations.[18]–[20].

The aim of this study was to assess the external validity of the initially reported diagnostic algorithm for the diagnosis of active pulmonary and extra-pulmonary tuberculosis in a Central European cohort. In addition we aimed to establish improved screening algorithms by machine learning methodology. For this purpose we aimed to construct two models – one including all useful laboratory and clinical parameters, and another model relying entirely on clinical information. The development of a diagnostic algorithm based on clinical information only was judged to being particularly useful for low-income regions.

## Materials and Methods

### Study Design and Outcome Parameters

This study was designed as a cross-sectional study. The study population consisted of 439 patients with clinical suspicion for active tuberculosis. All patients attending as out- or in-patients the Department of Infectious Disease at the Vienna General Hospital, Medical University of Vienna, between October 2001 and June 2008 were considered eligible, if the treating physician had requested laboratory testing of any biologic samples for mycobacterial culture.

Cases were classified as suffering from active tuberculosis by either a positive culture result for *M. tuberculosis* or a diagnosis based on either histology or radiology results suggestive for active tuberculosis and clinical cure following administration of specific anti-tuberculosis treatment. Non-tuberculosis patients were defined as subjects for whom biological samples had been sent for mycobacterial culture, but for whom an alternative diagnosis was established. Patients with HIV infection and paediatric patients were excluded from further analysis.

Patients being evaluated for tuberculosis routinely underwent assessment of serum inflammation markers at our institution. Those individuals for whom no results for acute phase parameters were available were excluded from further analysis. The inflammation parameters C-reactive protein, serum amyloid A, fibronectin, haptoglobin, and interleukin 6 were assessed routinely by nephelometry (Siemens DADE BN II). Similarly haematology, clinical biochemistry and blood sedimentation rates were performed routinely. Neopterin and transthyretin were analysed for the purpose of this study by ELISA (neopterin, Enzyme Linked Immunoassay, IBL Hamburg, Germany) and nephelometry (transthyretin, Siemens DADE Behring BN II) using frozen serum samples. Clinical information, microbiologic culture results, and results of histopathology and radiology were obtained from electronic patient records.

### Ethics Statement and Statistical Analysis

All participants provided written consent for the use and analysis of data and archived specimens. The study protocol was approved by the Ethics Committee of the Medical University of Vienna (EK: 724/2007). All data were pseudonymized and were entered into an electronic database and statistical analysis was performed using a commercially available software package (SPSS Statistics 16.0, SPSS Inc.). For comparison between groups Pearson’s χ^2^-test or a Mann-Whitney-U-test was applied as appropriate. Statistical significance was defined at a level of *α* = 0.05 and the Bonferroni-Holm approach was used for correction for multiple testing. For the purpose of validating a previously published diagnostic algorithm, outcome information was masked and data were sent for outcome prediction to the trial statistician of the previous study. [15] Classification of patients was performed by the blinded statistician and the outcomes were returned for the evaluation of the diagnostic accuracy for this data set. Further analysis was performed using various supervised machine learning techniques. We applied different such methods for classifying the feature based data into classes (TB, not TB), as desired.

Briefly describing the used methods, (I) a support vector machine (SVM) generates a discriminant function from training samples, based on so-called support vectors, maximizing the margin between classes. [21] Furthermore, (II) the ADTree + AdaBoost algorithm iteratively improves “weak” decision trees to a “strong” model, i.e., focusing on those instances that were misclassified in the previous iteration. [22] Furthermore, different prediction models were established using the (III) naïveBayes algorithm, calculating prior-, conditional- and posterior-probabilities, (IV) the logistic regression classifier, characterized by membership function for each class, and (V) the multi-layer perceptron (an artificial neural network), combining various linear models for non-linear classification.[21], [23]–[25] In this context attribute evaluators serve the purpose to skip irrelevant parameters of the data set prior to classification. Further optimization was performed by the discretization filter that converts continuous to nominal values and the principal components analysis (PCA), which transforms conceivably correlated parameters to an uncorrelated set of variables (i.e., transforms the variables to a different space, using the principal components as basis).[23], [26]–[28].

The Java based software suite WEKA (Waikato Environment for Knowledge Analysis, version 3.6.2, URL: http://www.cs.waikato.ac.nz/ml/weka/,licensed under GNU General Public License) was applied for the construction of improved diagnostic algorithms. [29] Missing values were not imputed in our data set. Optimization results of the models were assessed in internal validation. All training sets were trained with all major supervised classifying algorithms, maximizing the accuracy. When equal accuracy was rated, better Receiver Operating Characteristic (ROC) curve was used as selection criteria. [30] The outcome of the machine learning process was evaluated in a stratified 10-fold cross validation. [31], [32].

## Results

Following inclusion and exclusion criteria on all subjects being consecutively screened for tuberculosis a study population of 206 patients was constituted. 233 patients were excluded from further analysis, due to the unavailability of stored blood specimens (172 patients), missing data or loss of follow up (35 patients), diagnostic uncertainty or patients already receiving tuberculostatic therapy at the time of first physician’s contact (18 patients), infection with Mycobacteria other than tuberculosis (MOTT, 3 patients), HIV infection (4 patients) and age (1 patient).

Among those individuals 36 had a definitive diagnosis of active tuberculosis and 170 patients were suffering from other conditions (see: table 1). Distribution of diagnostic test for establishing diagnosis of active tuberculosis is presented in table 2. Clinical and laboratory characteristics of the study population are depicted in table 3. Median age, body mass index, C-reactive protein, serum amyloid A and were all significantly lower in tuberculosis than in non-tuberculosis patients in univariate analysis after adjustment for multiple testing using the Bonferroni-Holm procedure.

**Table 1:** Baseline characteristics of study population.

Type of disease	N	Group-Percentage
Auto-immune disease	11	6%
FUO	28	17%
Airway infection	64	38%
Abdominal infection	7	4%
Abscess	5	3%
Bone and joint-infection	3	2%
Soft tissue or foreign body-infection	4	2%
Endocarditis, pericarditis	9	5%
Neoplasm	35	21%
Other	4	2%
Total	170	100%
**Type of tuberculosis**		
Pulmonary TB	18	50%
Extra-pulmonary TB	15	42%
Miliary TB	3	8%
Total	36	100%

FUO =  fever of unknown origin, TB =  tuberculosis.

**Table 2:** Type of confirmation of active tuberculosis.

Detection method^1^	N	Group-Percentage
Clinically proven^2^	5	14%
Microscopy	2	6%
Histology	9	25%
PCR proven	5	14%
Culture proven	15	42%
Total	36	100%

1 classification into one category based on hierarchical evidence: culture, PCR, histology, microscopy, clinical prove;

2 with adequate response to therapy, PCR =  Polymerase Chain Reaction, IGRA =  Interferon Gamma Release Assay.

**Table 3:** Clinical and laboratory characteristics of tuberculosis and non-tuberculosis patients.

		Non-tuberculosis group (N = 170)		Tuberculosisgroup (N = 36)		
		N	%	N	%	p-value^1^
Male		103	61%	16	44%	0.075
Weight loss		69	41%	12	32%	0.596
Night sweat		64	39%	11	30%	0.671
**parameter**	**cut off** **	**median**	**IQR**	**median**	**IQR**	**p-value2**
Age		54	27	36	26	0.000*
C-reactive protein mg/l	<0.02	6	12	1	4	0.000*
Serum amyloid A mg/dl	<3.9	164	372	38.5	139	0.001*
Body mass index kg/m^2^	n.l.	23.2	5.9	19.1	5.2	0.005*
Mean corpuscular volume fl	n.l.	86.8	8	83.4	8	0.017
White blood count G/l	n.l.	7.9	5.4	6.1	2.8	0.017
Interleukin-6 pg/dl	<7	7	24	4	7	0.027
Haptoglobin mg/dl	<12	242	161	173	211	0.044
Temperature °C	n.l.	38	1.7	37.4	2	0.150
BSR^3^ 2 h mm	n.l.	80	40	72	38	0.151
Neopterin nmol/l	<1.35	11.7	19.6	8.8	13.7	0.183
BSR^3^ 1 h mm	n.l.	68	44	60	39	0.186
Transthyretin mg/dl	<5	16.1	12	17.3	13	0.243
Fibronectin mg/dl	<15	32	15	29	13	0.285
Hemoglobin g/dl	n.l.	12.2	3.2	12.3	2.7	0.556

1 Pearsońs χ^2^-test, nominal scale: yes or no.

2 U-test, continuous scale.

3 BSR: blood sedimentation rate.

* Statistically significant after adjusting for multiple testing by Bonferroni-Holm correction.

** typical analytical sensitivity-lower boundary (test kit lot depending), n.l. =  no limit.

### Evaluation of Diagnostic Algorithm

The data set was masked for outcomes and sent to the authors of the previously published study for analysis. Predicted outcomes were used for computation of diagnostic accuracy of the diagnostic algorithm in our patient population. One patient had to be excluded in this evaluation due to missing transthyretin values. Predicted outcomes are depicted in table 4.

**Table 4:** Diagnostic performance of tested diagnostic algorithms.

	Model Prediction				Accuracy	Sensitivity	Specificity	AUC-ROC*
	Pos		Neg					
**Support vector machine (Agranoff model, SVM 1)^1^**								
True TB		7		29	54.2% (47.1%–61.1%)	19.4% (8.2%–36.0%)	61.5% (51.5%–71.0%)	–
True NonTB		65		104				
**ADTree + AdaBoost (Agranoff model, AD 2)^2^**								
True TB	21			15	42.0% (35.1%–49.0%)	58.3% (40.1%–74.5%)	38.5% (31.1%–46.2%)	–
True NonTB	104			65				
**Support vector machine (Agranoff model, SVM 1, without extrapulmonary TB)^3^**								
True TB	4			14	57.8% (50.3%–65.0%)	22.2% (6.4%–47.6%)	61.5% (53.8%–68.9%)	–
True NonTB	65			104				
**ADTree + AdaBoost (Agranoff model, AD 2, without extrapulmonary TB)^4^**								
True TB	11			7	40.6% (33.5%–48.1%)	61.1% (35.8%–82.7%)	38.5% (31.1%–46.2%)	–
True NonTB	104			65				
**Logistic regression 1 (Optimal Performance Algorithm)** 5								
True TB	15			21	85.9% (80.4%–90.3%)	41.7% (25.5%–59.2%)	95.3% (90.9%–98.0%)	0.78
True NonTB	8			162				
**Naive Bayes 1 (Optimal Performance Algorithm)** 6								
True TB	22			14	81.1% (75.0%–86.2%)	61.1% (43.5%–76.9%)	85.3% (79.1%–90.3%)	0.79
True NonTB	25			145				
**Logistic regression (Clinical Data Algorithm)** 7								
True TB	13			23	84.5% (78.8%–89.1%)	36.1% (20.1%–53.8%)	94.7% (90.2%–97.6%)	0.66
True NonTB	9			161				
**Multilayer Perceptor 2 (Clinical Data Algorithm)** 8								
True TB	11			25	84.5%(78.8%–89.1%)	30.6% (16.4%–48.1%)	95.9% (91.7%–98.3%)	0.7
True NonTB	7			163				

AUC-ROC = Area under the Receiver Operation Characteristic curve; pos = positive, neg = negative.

95% confidence intervals are computed according binominal formula of Clopper and Pearson [44].

1,2 N = 205;

3,4 N = 187, 18 patients excluded due to extrapulmonary TB;

5 N = 205, with discretization, including: age, body mass index, C-reactive protein, night sweat;

6 N = 205,with discretization, principal components analysis; including: age, body mass index, C-reactive protein, night sweat;

7 N = 205, with discretization, principal components analysis; including: age, body mass index, night sweat;

8 N = 205, with normalization, 4 hidden layer; including: age, body mass index, night sweat;

In summary, the Gaussian kernel based support vector machine model (SVM 1) yielded a moderate diagnostic accuracy of 54% (47%–61%) when applied to our patient population showing sensitivity and specificity of 19% (8%–36%) and 62% (52%–71%), respectively. The second evaluated model, the meta-classifier model (AD 2) reached a diagnostic test accuracy of 42% (35%–49%) sensitivity: 58% (40%–75%), specificity: 38% (31%–46%).

### Development of Extended Diagnostic Algorithms

We aimed to develop two new diagnostic models by a machine learning approach – one making use of all available parameters (“Optimal Performance Algorithm”) and an alternative restricted to the use of clinical parameters (“Clinical Data Algorithm”). Firstly, most potent feature sets were identified to maximize the performance of the classification model. The feature selection process was started with single attribute evaluators, combined with a ranker search. All standard single attribute evaluators led to similar results, identifying the following six parameters: age, body mass index, C-reactive protein, serum amyloid A, weight loss, and night sweat. In an additional step, attribute subset evaluators were used on the original feature set and age, body mass index, C-reactive protein, and serum amyloid A were identified as evaluators. These results were consistent with the univariate analysis of variables. Two training sets were created with the aim to obtain two distinct diagnostic algorithms. Firstly we aimed to maximize test performance by including all useful parameters. Secondly we intended to construct a model that entirely relies on clinical information and may therefore prove particularly useful in low-income regions lacking the infrastructure to perform laboratory analysis of inflammation markers.

We tested the parameter sets with principal component analysis, the entropy based discretization method of Fayyad and Irani and a combination of both methods. [27], [28] The approach resulting in the best outcome in a stratified 10-fold cross validation was chosen. These included the following attributes for the “optimal performance set”: age, body mass index, C-reactive protein, night sweat. The discretization method of Fayyad and Irani, which yielded into improved models in this training set, was not able to establish discrete counterparts of serum amyloid A. For the clinical data model the parameters age, body mass index, and night sweats were identified.

All major supervised machine learning techniques were applied and evaluated by an internal 10-fold cross validation. According to these results, a logistic regression based classifier, the Naïve Bayes algorithm and a multilayer preceptor were identified as superior in our data set. Logistic regression based classification was performed with the ridge estimator of leChessie and van Houwelingen to establish an improved diagnostic model. [33] Naïve Bayes was used in standard settings. The multilayer preceptor was performed in standard settings using 4 hidden layers. [29].

The “Optimal Performance Algorithm” evaluated those parameters with best data pre-processing performance. The logistic regression based classifier was enhanced by the use of the discretization filter, and the Naïve Bayes was improved by the application of principal component analysis and the discretization filter.

Employing these settings a diagnostic accuracy of 86% (80%–90%) was achieved for our patient population with an area under the curve (AUC) of the receiver operating characteristic (ROC) of 0.78. In this analysis the sensitivity was 42% (26%–59%) and the specificity was 95% (91%–98%). The true positive rate for tuberculosis cases in our study population was between 42% and 61% (see: table 4).

For the evaluation of the “Clinical Data Algorithm” the multilayer preceptor employing in standard settings showed the best accuracy. A diagnostic accuracy of 85% (79%–89%) could be achieved. Sensitivity [31% (16%–48%), specificity: 96%, (92%–98%)] and the AUC of the ROC curve (0.7) was lower than the previously established model. The logistic model combined with discretization and principal components analysis led to a similar result but to a lower ROC curve (see: table 4).

## Discussion

Rapid and reliable diagnostic tests for tuberculosis are urgently needed and the previously published diagnostic algorithms showed highly encouraging accuracy. Provided that this good diagnostic precision is reproducible for diverse patient populations and settings, such a rapid assessment tool, which could be part of a point-of-care test for active tuberculosis, would constitute a major improvement in the diagnosis, management, and control of tuberculosis. [34].

In this study the previously published diagnostic classification model was evaluated in a Central European patient population. The diagnostic accuracy was disappointingly low at 54% and 42%. This poor diagnostic performance may be explained by various factors. Whereas the analysis of data was identical in both studies – and classification of cases was performed by the same person as in the initial study – the patient population under investigation differed considerably between the two studies.

Whereas Agranoff and colleagues worked with a study population predominantly suffering from pulmonary tuberculosis, our study population included a significant proportion of patients with extra-pulmonary tuberculosis. However, in our data set the diagnostic accuracy of the Agranoff model was not significantly improved when restricting the analysis to only those individuals suffering from pulmonary tuberculosis [n = 19; accuracy: 58% (SVM) and 41% (AdaBoost) see: table 4].

Contrary to the previous study, no HIV seropositive patients were included in our study. Other potential differences may include variations in treatment seeking behaviour, diagnostic approaches of caring physicians, differences in ethnicity of patients, and a discrepancy in pre-test probability based on an unequal numeric distribution of cases and controls.

Whereas an equal number of cases and controls was selected in Agranoff´s study following a case-control study design, we used a cross-sectional study design applying predefined inclusion and exclusion criteria in order to avoid an artificially high proportion of tuberculosis patients in our data set. Therefore all consecutive patient with clinical suspicion for tuberculosis were included leading to a 1∶4 distribution of tuberculosis and non-tuberculosis patients, respectively. Differences in the pre-test probability invariably affect the performance of diagnostic models and may be an explanation of impaired generalizability of both the previously published and the newly established model.

In addition, the ethnic origin of patients was unevenly distributed in Agranoff’s training set. Whereas 79% of tuberculosis patients originated from sub-Saharan Africa in the training set, the proportion Africans was only 34% in the control group. Furthermore the control group in that study was heterogeneous consisting of both patients suffering from inflammatory conditions and healthy volunteers. Whereas limitations of our study are the retrospective identification of this patient cohort, a limited sample size, and exclusion of potential participants due to missing data for a proportion of identified subjects, a great emphasis was laid on the constitution of a homogenous comparator that was entirely chosen based on the exposure (suspicion for tuberculosis) and not for the outcome under investigation (diagnosis of tuberculosis). All these factors may explain the lower than expected diagnostic accuracy of the initially published model and stress the need for further improvement and prospective evaluation of this diagnostic algorithm in various clinical settings.

Following our goal to develop an improved diagnostic algorithm, we used machine learning methodology to obtain an improved diagnostic algorithm. The “Optimal Performance Algorithm”, including age, body mass index, night sweat, C-reactive protein led to a diagnostic accuracy of 86% (80%–90%) with an AUC of the ROC-curve of 0.78 in an internal 10-fold cross validation. Similarly the “Clinical Data Algorithm”, consisting of age, body mass index and night sweat, had a diagnostic accuracy of 85% (79%–89%) and an AUC-ROC of 0.70. Considering the ease of obtaining the respective clinical parameters and the variability in the model estimation the Clinical Data Algorithm seems particularly useful. This finding may also be interpreted in that way that the inclusion of inflammation parameters does not significantly improve diagnostic models in tuberculosis. However further prospective evaluation in these diverse clinical settings and comparative evaluation to the diagnostic accuracy by a skilled physician is warranted in future prospective studies.

Considering the presented results, no final judgment may therefore be given whether machine learning based diagnostic algorithms are an appropriate screening method for tuberculosis or not. Arguably clinical parameters of patients suffering from tuberculosis may vary considerably and other parameters than inflammation parameters may prove more suitable as markers for the screening of patients. These markers may include serum concentrations of calcium [35]–[37], iron [38], vitamin D [39]–[41] or orosomucoid [42], [43] and it may prove rewarding to evaluate those alone or in combination in future diagnostic algorithms.

In summary this study demonstrates low external validity of the previously published machine learning based diagnostic algorithm when evaluated for our patient population. Although diagnostic algorithms with improved diagnostic precision were established based on data of a Central European patient population, further independent prospective evaluation of these models is needed to better appreciate the potential of machine learning based diagnostic algorithms for the rapid screening of patients for active tuberculosis.
